# SecY-mediated quality control prevents the translocation of non-gated porins

**DOI:** 10.1038/s41598-020-73185-y

**Published:** 2020-10-01

**Authors:** Sebastian Jung, Verian Bader, Ana Natriashvili, Hans-Georg Koch, Konstanze F. Winklhofer, Jörg Tatzelt

**Affiliations:** 1 Department Biochemistry of Neurodegenerative Diseases, Institute of Biochemistry and Pathobiochemistry, Ruhr University Bochum, Universitätsstr. 150, 44801 Bochum, Germany; 2 Institute of Biochemistry and Molecular Biology, ZBMZ, Faculty of Medicine, Albert-Ludwigs-University Freiburg, Freiburg im Breisgau, Germany; 3 Department Molecular Cell Biology, Institute of Biochemistry and Pathobiochemistry, Ruhr University Bochum, Bochum, Germany; 4 Faculty of Biology, Albert-Ludwigs-University Freiburg, Freiburg im Breisgau, Germany

**Keywords:** Cell biology, Cell biology, Molecular biology, Molecular biology

## Abstract

OmpC and OmpF are among the most abundant outer membrane proteins in *E. coli* and serve as hydrophilic channels to mediate uptake of small molecules including antibiotics. Influx selectivity is controlled by the so-called constriction zone or eyelet of the channel. Mutations in the loop domain forming the eyelet can disrupt transport selectivity and thereby interfere with bacterial viability. In this study we show that a highly conserved motif of five negatively charged amino acids in the eyelet, which is critical to regulate pore selectivity, is also required for SecY-mediated transport of OmpC and OmpF into the periplasm. Variants with a deleted or mutated motif were expressed in the cytosol and translocation was initiated. However, after signal peptide cleavage, import into the periplasm was aborted and the mutated proteins were redirected to the cytosol. Strikingly, reducing the proof-reading capacity of SecY by introducing the PrlA4 substitutions restored transport of OmpC with a mutated channel domain into the periplasm. Our study identified a SecY-mediated quality control pathway to restrict transport of outer membrane porin proteins with a deregulated channel activity into the periplasm.

## Introduction

A characteristic feature of Gram-negative bacteria is an outer membrane (OM) in addition to the cytoplasmic/inner membrane (IM). The OM has crucial functions in protecting the cell against environmental threats and in regulating influx of nutrients and excretion of cellular products. Almost all of the transmembrane proteins in the OM are β-barrel proteins. Among them are the outer membrane porin proteins (OMPs), such as OmpC and OmpF, that form water-filled channels for the passage of a large variety of hydrophilic molecules including antibiotics (rev in^1^). Pore diameter and influx selectivity is controlled by the so-called loop L3 between β-strands 5 and 6, which folds back into the pore from the extracellular side of the OM and builds a constriction zone or eyelet within the porin. Pore selectivity is regulated by a highly conserved motif of negatively charged amino acids in the eyelet that interacts with basic amino acids, mostly Arg, located in the wall of the porin^2–4^. The findings that mutations in this motif can alter pore selectivity and transport of antibiotics support the concept that OMPs are attractive targets for antibiotic therapies^5–9^.

Like most of the outer membrane proteins, OmpC and OmpF are post-translationally translocated across the inner membrane by the conserved Sec translocon^10^. The bacterial Sec translocon consists of three core proteins, SecY, SecE and SecG^11,12^, which associate with multiple partner proteins for facilitating the transport of a large variety of different secretory and inner membrane proteins^13^. The protein conducting channel is formed by the SecY subunit, which consists of 10 transmembrane domains (TMs) that create two water-filled cavities, separated by a central constriction in the middle of the membrane^14^. This constriction is called the pore ring and is composed of six hydrophobic isoleucine residues in *E. coli*. The pore ring together with a short helix, called the plug, is supposed to prevent uncontrolled ion leakage across the resting translocon^15^. Movements of TMs 2B, 3, 7 and 8 during protein transport cause the formation of a crevice, called the lateral gate that allows membrane protein substrates to exit the SecY channel into the lipid phase^16^. The dynamic structure of SecY is stabilized by the SecE subunit^17^, which is located on the back of the SecY channel, opposite of the lateral gate^14^. The SecG subunit is not essential for protein transport in bacteria^18^ and has been replaced by the non-homologous Sec61β subunit in archaea and eukaryotes. This probably reflects its functional interaction with the ATPase SecA, which is absent in archaea and eukaryotes and which serves as SecY-associated receptor for secretory proteins in bacteria^19^. SecA serves a dual function during transport of secretory proteins such as OmpC or OmpF: SecA specifically recognizes the N-terminal signal sequence of its client proteins and provides together with the proton motive force the energy for translocation by repetitive ATP hydrolysis cycles^20, 21^. The signal sequence is likely transiently trapped at the lateral gate^22^ which provides a lever for the ATP driven stepwise substrate translocation by SecA^23,24^. The intricate structure of the SecY channel also allows for a proof-reading activity, which is impaired in the *prlA* mutants of SecY^25^. The *prlA* alleles allow the translocation of secretory proteins with defective signal sequences or even when a signal sequence is missing. The *prlA* mutations all localize to the pore ring, the plug residues, the lateral gate and the channel interior and they are considered to stabilize the open state of the SecY channel^26^.

After translocation the OMPs are kept in an unfolded state by the periplasmic chaperones survival protein A (SurA) and seventeen kilodalton protein (Skp) and are guided to the beta-barrel assembly machinery (BAM). The BAM complex with its core protein BamA finally inserts the OMPs into the OM (rev in^27,28^). To prevent accumulation of folding-incompetent OMPs in the periplasm, misfolded or aggregated conformers are either degraded by DegP or secreted via outer membrane vesicles (OMVs)^29,30^. Surprisingly, little is known about quality control pathways that operate during SecY-mediated translocation in order to prevent transport of aberrant OMPs into the periplasm. Here we show that such a quality control mechanism exists. OmpC and OmpF variants with mutated pore residues are subjected to a SecY-mediated quality control pathway. As a consequence, translocation is initiated but aborted after signal peptide cleavage.

## Results

### A conserved alpha-helical domain is required for periplasmic localization of OmpC and OmpF

**Figure 1:**
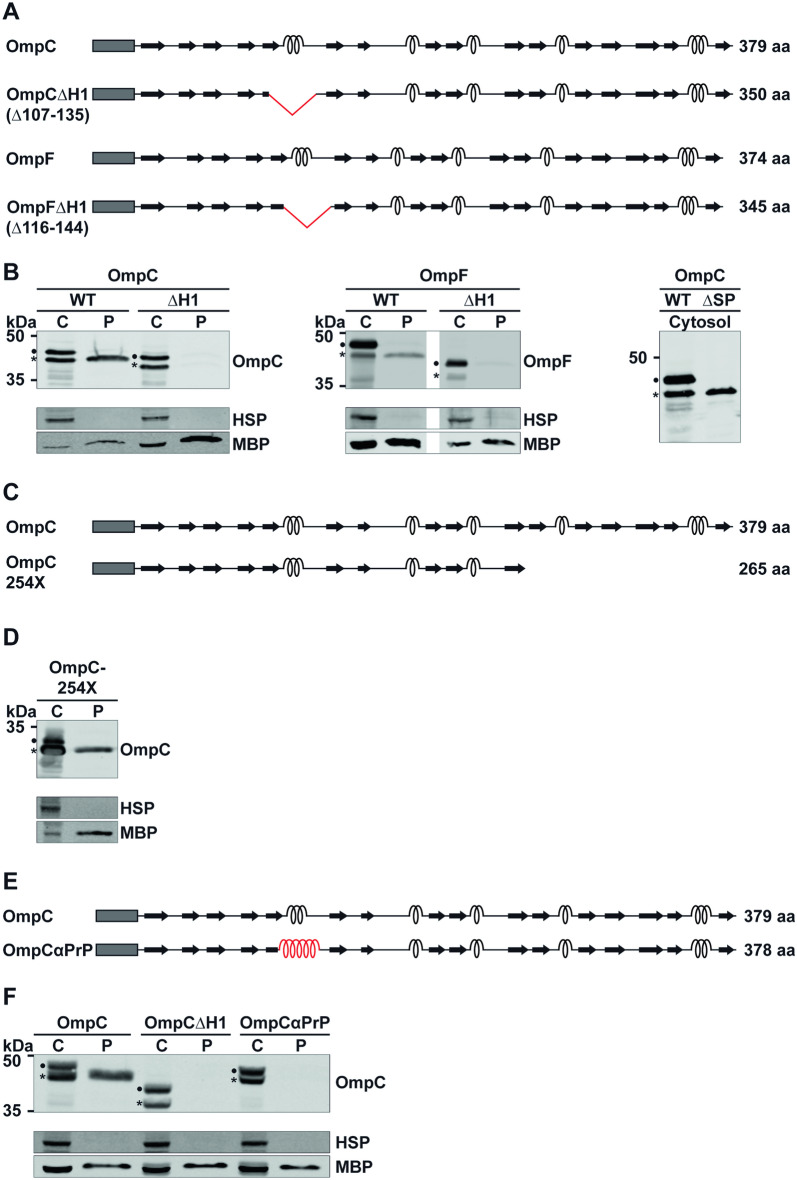
A conserved alpha-helical domain is critically required for periplasmic localization of OmpC and OmpF. (**A**,**C**,**E**) Schematic presentation of the constructs analyzed. Dark rectangle: signal peptide; arrows indicate beta-strands, α-helical structure is indicated by helices, the α-helical domain derived from PrP is shown in red (E); amino acids deleted are shown by red triangle and listed below protein name. The total length of the proteins is indicated. (**B**,**D**,**F**) Transformed *E. coli* cells expressing the proteins indicated were fractionated and the cytoplasmic (C) and periplasmic (P) fraction analyzed by Western blotting. Unprocessed full length constructs (●) and constructs after signal peptide cleavage (*) are marked. Cytosolic heat shock proteins (HSP) and the periplasmic maltose-binding protein (MBP) were analyzed in parallel. Uncropped images of representative Western blots can be found in Supplementary Figures 1 and 2.

We have previously shown that the efficiency of SecY- and Sec61-mediated translocation is regulated by the secondary structure of secretory proteins^31–35^. For example, model substrates composed of beta-strands only are less efficiently exported into the periplasm of *E. coli* than proteins dominated by alpha-helical domains. However, impaired translocation of beta-strands is restored by fusing them to alpha-helical domains^35^. Interestingly, beta-barrel proteins of the OM in *E. coli*, such as OmpC and OmpF, which are translocated via the SecYEG complex into the periplasmic space, contain several short alpha-helical domains in addition to beta-strands (UniProtKB P06996, P02931) (Fig. 1A). Thus, we were wondering whether these alpha-helical domains play a role in promoting translocation efficiency.

To address this hypothesis experimentally, we analyzed biogenesis of OmpC and OmpF variants lacking the first alpha-helical domain (Fig. 1A). The rationale for targeting helix1 was based on the finding that alpha-helical domains close to the signal peptide have the highest efficiency in increasing translocation^31,33–35^. SecY-mediated transport in *E. coli* was analyzed by separating transformed bacteria into periplasmic and cytoplasmic fractions and subsequent Western blotting. To monitor purity of the periplasmic fraction, cytosolic heat shock proteins (HSP) and the periplasmic maltose-binding protein (MBP) were analyzed in parallel. Usually, minor amounts of MBP are found in the cytosolic fraction, since this fraction also contains *E. coli* with an incompletely permeabilized outer membrane. However, the absence of HSPs in the periplasmic fraction indicates that this fraction is devoid of cytoplasmic proteins. As expected, wildtype (wt) OmpC and OmpF were present in the periplasm (Fig. 1B). In contrast, no significant amounts of OmpC or OmpF variants lacking helix1 (OmpC∆H1, OmpF∆H1) were detectable in the periplasmic fraction. This phenomenon was not caused by decreased synthesis or degradation in the cytosol since both variants were present in the cytoplasmic fraction in amounts comparable to the wt proteins (Fig. 1B). Notably, the cytosolic fraction of both wt and ∆H1 proteins consisted of two distinct species. Expression of an OmpC variant lacking the signal peptide (OmpC∆SP) indicated that the faster migrating species represents a fraction processed by the signal peptidase (Fig. 1B, ∆SP, right panel).

To study whether impaired periplasmic localization of OmpC∆H1is a general phenomenon of OmpC variants bearing a deletion, we created a different variant with a large deletion in the C-terminal domain (OmpC-254X, Fig. 1C). In contrast to OmpC∆H1, OmpC-254X was exported into the periplasm (Fig. 1D), indicating that the presence of α-helix1 appears to be a specific determinant for OmpC translocation. The activity of helix1 to promote periplasmic localization may reside in its primary sequence and/or its ability to adopt an alpha-helical structure. To address a possible role of the secondary structure, we performed domain-swapping experiments and replaced helix1 of OmpC by an alpha-helical domain derived from the prion protein^36^ (Fig. 1E). Notably, the potent activity of this alpha-helical domain to promote Sec61- and SecY-mediated translocation of proteins composed of beta-sheets was demonstrated previously^35^. Surprisingly, the heterologous alpha-helical domain failed to increase periplasmic localization of OmpC lacking helix1. Similarly to OmpC∆H1, OmpCαPrP was only detectable in the cytosolic fraction, indicating a crucial role of the primary sequence of helix1 in promoting translocation into the periplasm (Fig. 1F).

### Periplasmic export of OmpC with a mutated eyelet is aborted after initiation of translocation

**Figure 2:**
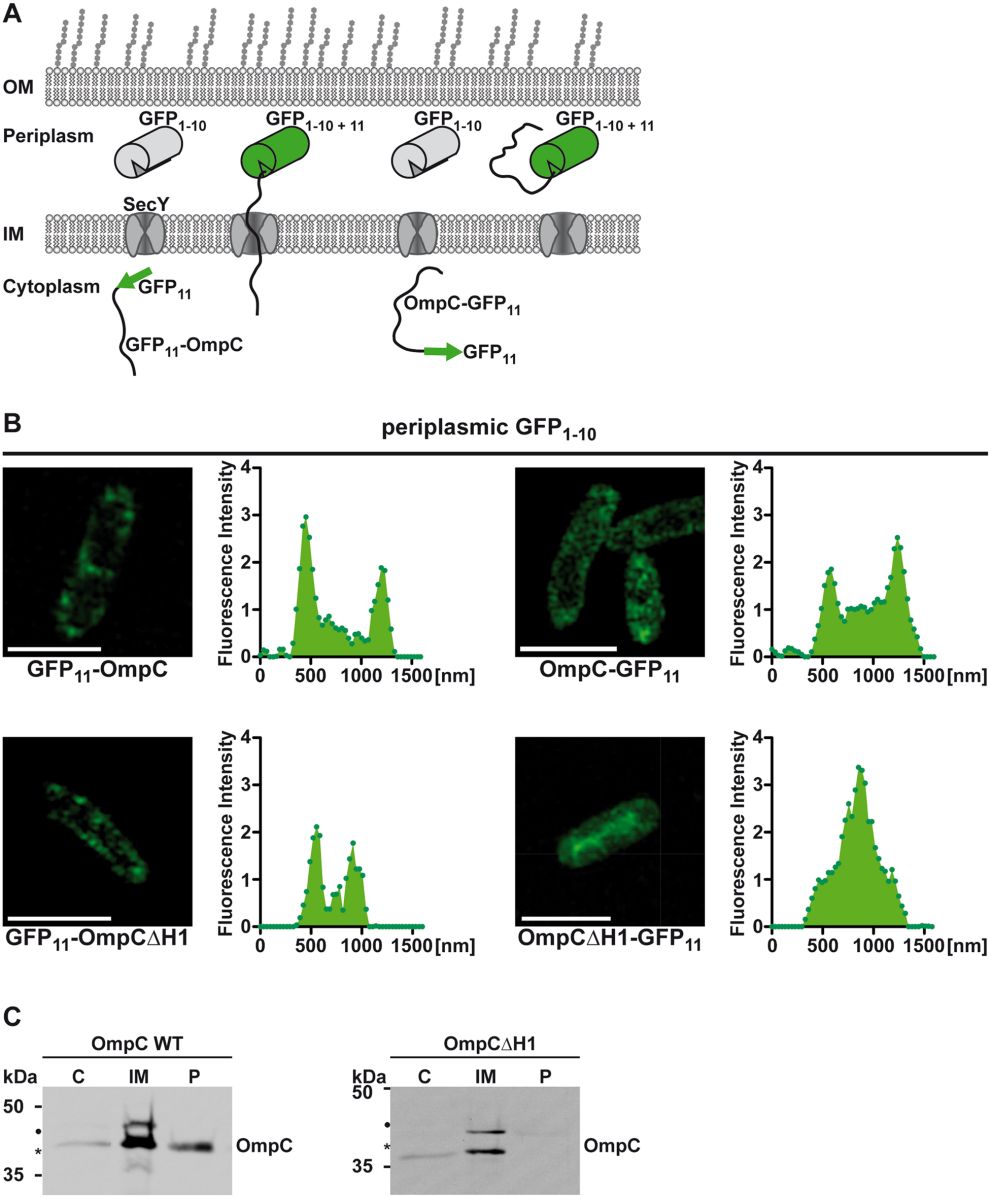
Periplasmic export of OmpC with a mutated eyelet is aborted after initiation of translocation. (**A**) Schematic representation of the in vivo complementation assay. The 11th beta-strand of superfolder GFP (GFP_11_) was fused to either the N- or C-terminus of OmpC or OmpC(∆H1). GFP_1-10_, composed of the first 10 beta strands of GFP, was targeted to the periplasm by a dsbA signal peptide. To achieve complementation and GFP fluorescence in the periplasm OmpC variants with a C-terminal GFP11 have to be completely imported into the periplasm. For GPF11 is at the N-terminus a partial translocation is sufficient for obtaining a fluorescent signal. (**B**) Super-resolution structured illumination microscopy (SR-SIM) images from *E. coli* cells co-expressing the indicated proteins, scale bar is 2 µm. Fluorescence intensity histograms are shown next to respective SIM images. Histogram data were acquired as cross section through *E. coli* cells. (**C**) Subcellular fractionation by ultracentrifugation. Transformed *E. coli* cells expressing the proteins indicated were fractionated into cytoplasmic (C), inner membrane (IM), and periplasmic (P) fractions by sucrose gradient centrifugation and the respective fractions were analyzed by Western blotting. Unprocessed full length constructs (●) and constructs after signal peptide cleavage (*) are marked. Uncropped images of representative Western blots can be found in Supplementary Figure 2.

The Western blot analyses showed above indicated that the OmpC or OmpF variants lacking Helix1 are processed by the signal peptidase but not further translocated into the periplasm. Based on this observation, we tested the hypothesis that translocation of OmpC∆H1 is initiated by the SecYEG-translocon but then aborted after part of the N-terminal domain has been translocated into the periplasm. To this end, we made use of split GFP protein tagging^37^. We targeted a superfolder GFP 1–10 via the DsbA signal peptide to the periplasm and fused a GFP 11 tag to either the N- or C-terminus of OmpC. (Fig. 2A). In vivo complementation of GFP fluorescence was monitored in transformed *E. coli* by super-resolution structured illumination microscopy. Fluorescence intensity and subcellular distribution was analyzed using the Carl Zeiss ZEN Black software. As expected, co-expression of periplasmic GFP1-10 and GFP11-OmpC or OmpC-GFP11 resulted in green fluorescence in the periplasm of transformed *E. coli* (Fig. 2B, top panels). A fluorescence signal was also observed in cells after co-expression of periplasmic GFP1-10 and OmpC∆H1 fused to GFP11 at its N-terminus (GFP11-OmpC∆H1), indicating that the N-terminus is able to interact with the GFP1-10 moiety in the periplasm. In contrast, OmpC∆H1 tagged with C-terminal GFP11 (OmpC∆H1-GFP11) failed to complement periplasmic GFP1-10, but showed a fluorescence in the center of the cell, indicative of a cytosolic localization (Fig. 2B, lower panels). These experiments support a scenario in which OmpC∆H1 is targeted to and partially translocated through SecY but complete translocation is aborted after cleavage of the signal peptide. To support the results found by super-resolution microscopy, we fractionated *E. coli* cells expressing wildtype OmpC and OmpC∆H1 into cytoplasmic, inner membrane, and periplasmic fractions. Corroborating the previous results OmpC∆H1 was absent in periplasmic fraction. However, it was found in the inner membrane fraction, similarly to wildtype OmpC (Fig. 2C), supporting the concept of an arrested translocation.

### Helix1 promotes periplasmic localization of OmpC in a sequence-dependent manner

**Figure 3:**
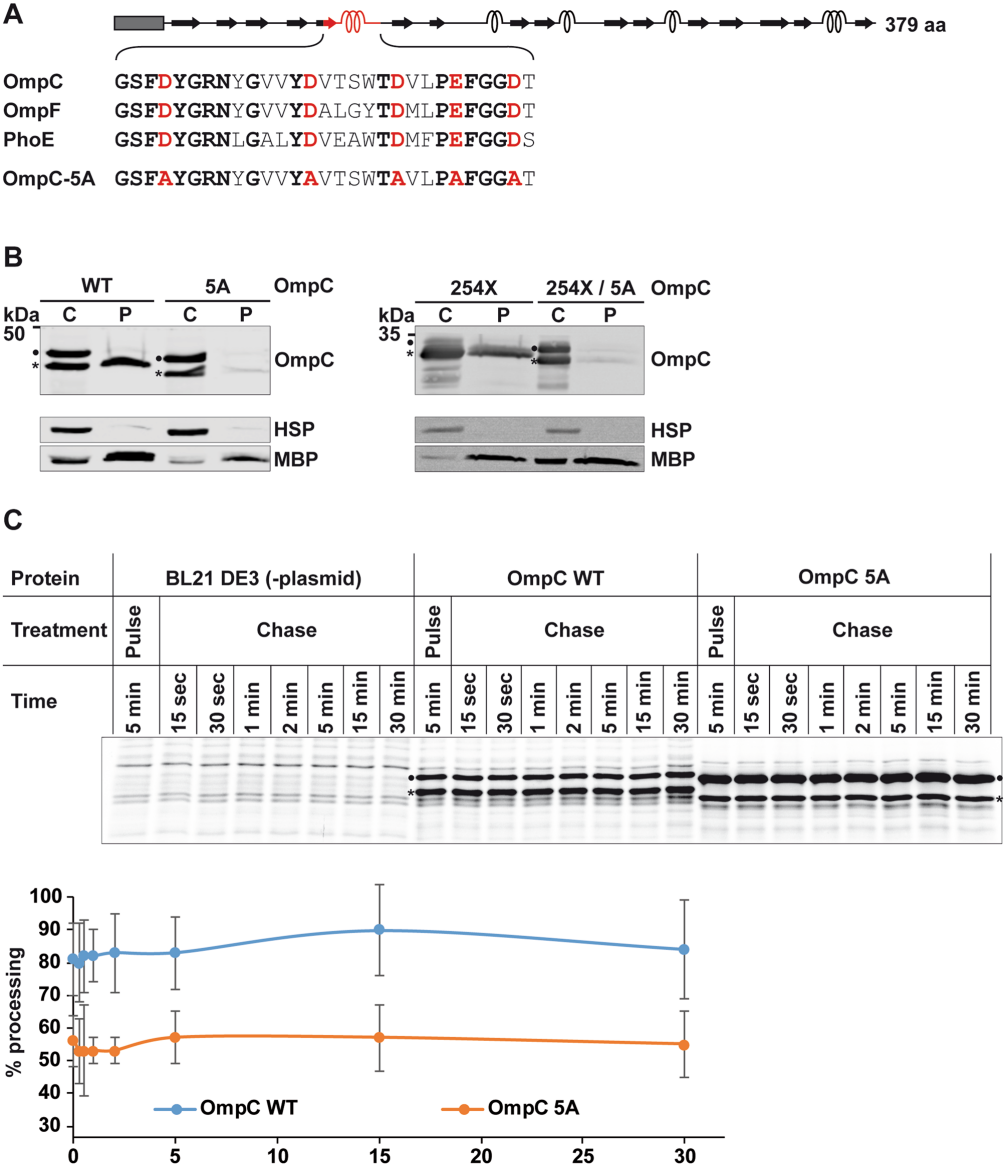
Helix1 of OmpC promotes periplasmic localization in a sequence-dependent manner. (**A**) Schematic presentation of the constructs analyzed. Dark rectangle: signal peptide; arrows indicate beta-strands, α-helical structure is indicated by helices. The amino acid sequences of the conserved motif of OmpC, OmpF and PhoE are shown in the detail magnification. Conserved residues are indicated in bold. The amino acid substitutions of the OmpC5-A variant are marked in red. The total length of the protein is indicated. (**B**) Transformed *E. coli* cells expressing the proteins indicated were fractionated and the cytoplasmic (C) and periplasmic (P) fraction analyzed by Western blotting. Unprocessed full length constructs (●) and constructs after signal peptide cleavage (*) are marked. Cytosolic heat shock proteins (HSP) and the periplasmic maltose-binding protein (MBP) were analyzed in parallel. Uncropped images of representative Western blots can be found in Supplementary Figure 3. (**C**) Pulse chase experiments with *E. coli* BL21 DE3 expressing the proteins indicated or transformed with empty pET27b vector as control (-plasmid). Cells were labeled with ^35^S methionine/cysteine after rifampicin addition, which blocks the endogenous RNA polymerase but not T7 RNA polymerase. After 5 min of pulse, excess non-radioactive methionine/cysteine was added and samples were taken at the indicated time points, separated by SDS-PAGE and analyzed by phosphor imaging. Statistics is shown for at least two biological replicates with two technical replicates each. The error bars represent the standard error of the mean (SEM). Uncropped images of Autoradiogram can be found in Supplementary Figure 3.

Helix1 of OmpC and OmpF forms the constriction zone or eyelet of the channel. It contains a highly conserved motif of five negatively charged amino acids present in OmpC, OmpF and PhoE (Fig. 3A, marked in red), which is critical to regulate pore selectivity^2^. Indeed, single missense mutations of these residues can affect transport of antibiotics and strain susceptibility (Ref. in^38^). To analyze a possible role of the conserved acidic side chains, we mutated the four aspartates and the one glutamate to alanines (OmpC-5A) (Fig. 3A). Remarkably, removing these negative charges from the conserved motif had the same effect as deleting helix1. Similarly to OmpC∆H1, the OmpC-5A variant was found in the cytosol but was barely detectable in the periplasmic fraction (Fig. 3B, left panel). Please note that the replacement of the negative charges by alanin slightly changes the running behaviour on SDS-PAGE. The pivotal role of the negatively charged motif in promoting import of OmpC into the periplasm was further demonstrated by introducing the 5A substitution into OmpC-254X. While OmpC-254X was present in the periplasm, the OmpC-254X/5A variant could be detected only in the cytosolic fraction (Fig. 3B, right panel).

For both OmpC∆H1 and OmpC-5A we noticed that signal sequence was cleaved without complete translocation into the periplasm. This indicated that the signal sequence was inserted into the SecYEG channel and the signal peptidase cleavage site was accessible to the periplasmically localized catalytic domain of signal peptidase^10^. This was further validated by pulse labeling in vivo. *E. coli* cells carrying T7 RNA polymerase-dependent expression plasmids for either wildtype OmpC or OmpC-5A were grown on minimal media lacking methionine and cysteine and protein production was induced by IPTG. After further incubation, endogenous transcription was blocked by the addition of rifampicin and ^35^S-labeled methionine/cysteine was added (pulse). After 5 min of labeling, excess non-radioactive methionine/cysteine (chase) was added and samples were withdrawn at different time points over a period of 30 min. For both wildtype OmpC and the OmpC-5A variant, signal sequence cleavage was already observed after 15 s of chase (Fig. 3C). Although processing of OmpC-5A was lower than for wildtype OmpC, we did not notice any obvious difference in the kinetics of signal sequence cleavage (Fig. 3C, lower panel). This provides further indication that OmpC-5A is processed by signal peptidase, but fails to be completely translocated into the periplasm (Fig. 3B). The reduced processing of OmpC-5A in the pulse-labeling experiment could reflect that some SecYEG channels are jammed by the non-completely translocated OmpC-5A variant.

**Figure 4:**
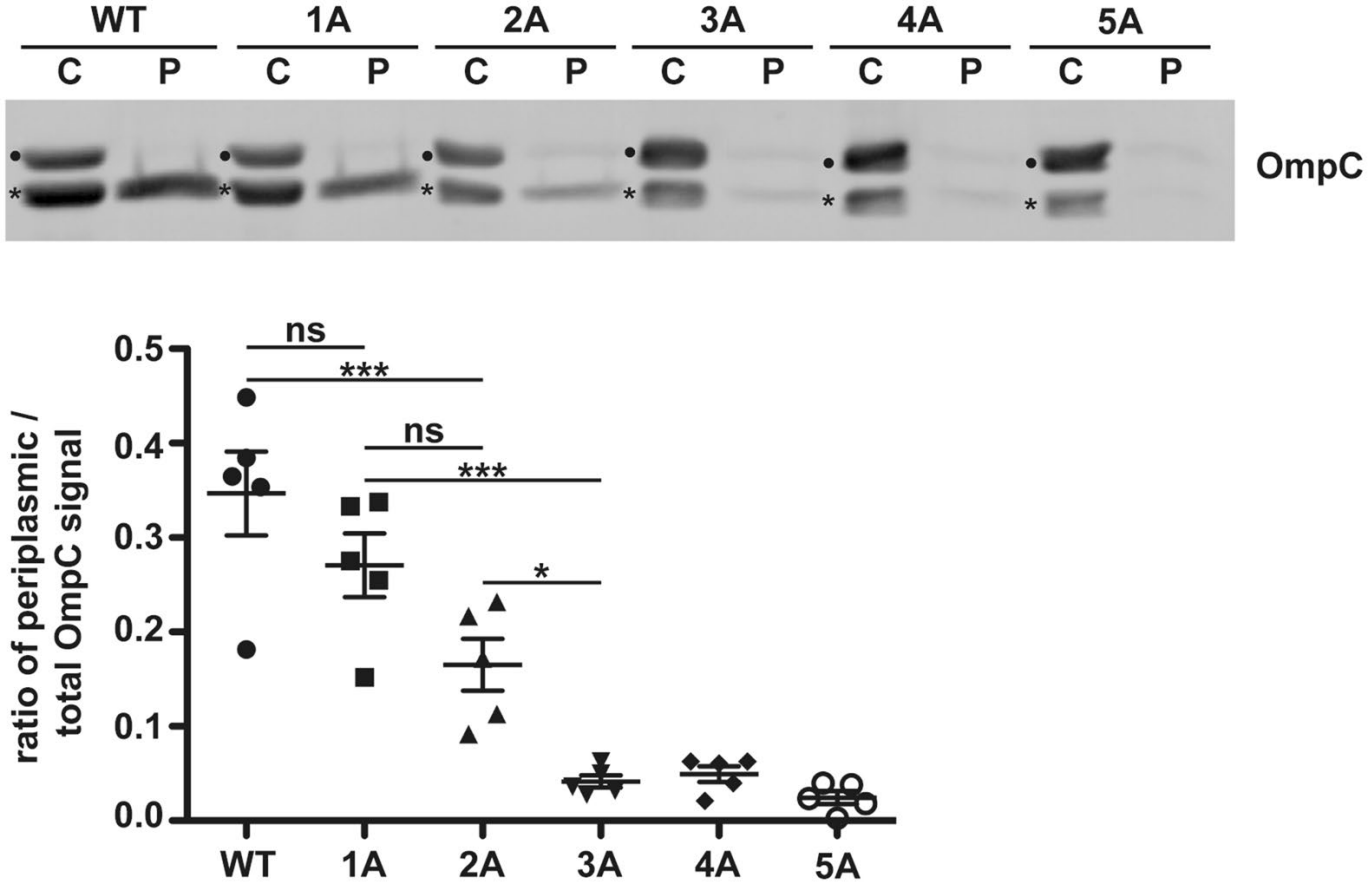
SecY-mediated translocation of OmpC is dependent on the net charge of its conserved alpha helical domain. Transformed *E. coli* cells expressing the proteins indicated were fractionated and the cytoplasmic (C) and periplasmic (P) fraction analyzed by Western blotting. Unprocessed full length constructs (●) and constructs after signal peptide cleavage (*) are marked. Uncropped image of representative Western blot can be found in Supplementary Figure 4. Data represent the mean plus/minus SEM from n = 5 biological replicates. To analyze the distribution of datasets (parametric/non-parametric), Kolmogorov–Smirnov test were performed. For the comparison of more than two parametric datasets, one-way ANOVA was used. To correct for α-error inflation resulting from multiple comparisons, ANOVA Dunnett’s post hoc multiple comparison test was performed.

These experiments indicated that periplasmic localization of OmpC is critically dependent on the highly conserved negatively charged motif in the eyelet, which regulates pore selectively. To study the role of the charged amino acids for translocation we generated new variants in which the negatively charged residues were substituted with alanines one by one: 1A (D110A), 2A (D110A, D120A), 3A (D110A, D120A, D126A), and 4A (D110A, D120A, D126A, E130A). We analyzed translocation efficiency of those variants compared to wildtype OmpC and the 5A variant used before (Fig. 4). We saw a gradual decrease in translocation efficiency until the first three Ds were substituted by As. Interestingly, no further decrease was seen by removing the remaining two negatively charged amino acids, indicating that a certain net charge of the domain is necessary to maintain translocation competence of OmpC.

### The proof-reading activity of SecY prevents translocation of OmpC with a mutated pore domain

**Figure 5:**
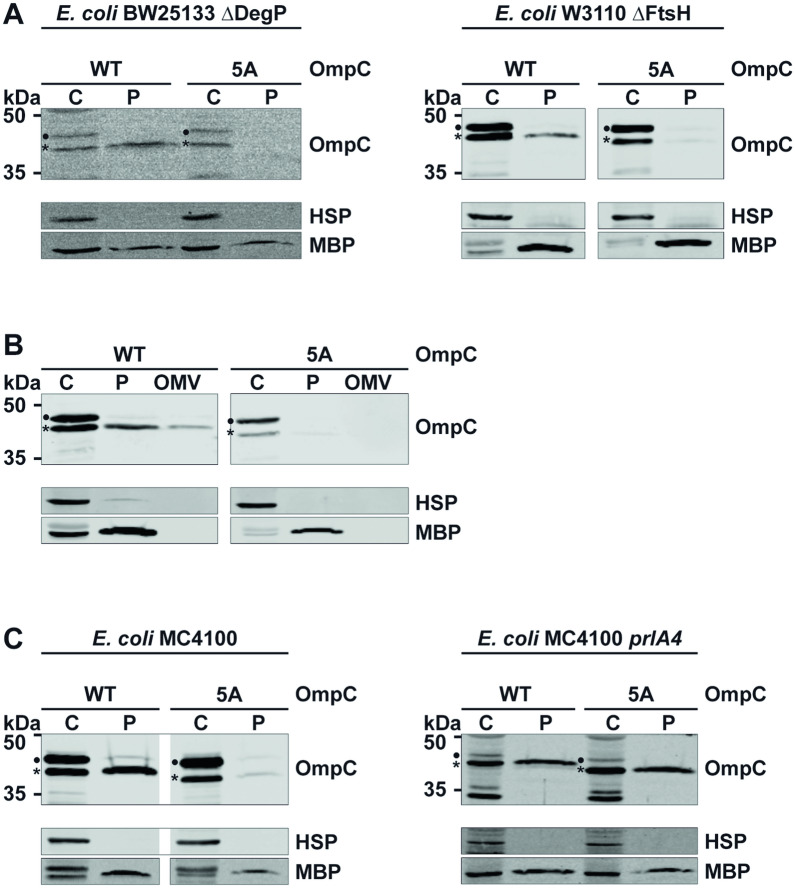
A proof-reading activity of SecY prevents translocation of OmpC with a mutated pore domain. (**A–C**) Transformed *E. coli* cells expressing the proteins indicated were fractionated and the cytoplasmic (C) and periplasmic (P) fraction analyzed by Western blotting. Unprocessed full length constructs (●) and constructs after signal peptide cleavage (*) are marked. Cytosolic heat shock proteins (HSP) and the periplasmic maltose-binding protein (MBP) were analyzed in parallel. (**A**) The indicated constructs were expressed in *E. coli* mutants devoid of either the periplasmic protease Degp (left panel) or FtsH, an integral membrane protease of the inner membrane (right panel). (**B**) Outer membrane vesicles (OMV) were prepared from *E. coli* cells expressing OmpC or OmpC-5A and analyzed by Western blotting. The cytosolic (C) and periplasmic (P) fractions were analyzed in parallel. (**C**) Expression of OmpC and OmpC-5A was analyzed by Western blotting in an *E. coli* mutant strain harboring the *prlA4* mutation in SecY (right panel). The corresponding wildtype strain (MC4100) was analyzed in parallel (left panel). Uncropped images of representative Western blots can be found in Supplementary Figures 5 and 6.

To provide additional experimental support for the concept that OmpC variants with a mutated pore domain are not completely translocated, we analyzed two previously described quality control pathways for misfolded OMPs in the periplasm: degradation by DegP^39^ or FtsH^40^ and secretion via outer membrane vesicles (OMVs)^30^. First, we analyzed OmpC-5A in *E. coli* strains lacking DegP or FtsH. In case OmpC-5A is degraded by one of these proteases, it should now be stabilized in the periplasm. However, OmpC-5A was not detectable in the periplasmic fractions of both DegP- and FtsH-deficient strains (Fig. 5A). To address a possible vesicular secretion, we analyzed OMVs prepared from *E. coli* expressing wildtype OmpC or OmpC-5A. Whereas a fraction of wildtype OmpC was present in these vesicles the 5A variant was not secreted via this pathway (Fig. 5B). These results also show that OmpC-5A is not inserted into the outer membrane. Next, we addressed the possibility that the conserved motif in the eyelet is required for efficient transport of OmpC through SecY. To this end we made use of the *prlA4* strain of *E. coli*^25^. In this strain two amino acid substitutions in SecY (F286Y and I408N)^41^ enable translocation of pre-proteins with a defective or missing signal sequence^42–44^. In previous studies it was concluded that the amino acid substitutions in transmembrane segment 7 (F286Y) and 10 (I408N) prevent the rejection of defective pre-proteins from the export pathway^45^ by promoting a translocation-competent conformation of the translocon in the absence of a signal peptide^46,47^. Since the *prlA4* strain has a different genetic background we first recapitulated the impaired translocation of OmpC-5A in the corresponding wildtype *E. coli* strain MC4100. Also in this strain only wildtype OmpC, but not OmpC-5A was detectable in the periplasmic fraction (Fig. 5C, left panel). However, translocation of OmpC with a mutated eyelet into the periplasm was restored by reducing the proof-reading activity of SecY. Similarly to wildtype OmpC, OmpC-5A was found in the periplasmic fraction of the *prlA4* strain (Fig. 5C, right panel). For wildtype OmpC basically no pre-from of OmpC was seen, so processing and transport is enhanced even for wildtype by the prlA4 mutant.

## Discussion

To maintain integrity and functionality of the OM, it is crucial to prevent accumulation of non-natively folded proteins in the periplasm and to ensure cargo specificity of the transport systems spanning the OM. A major challenge of this task is the lack of ATP in the periplasm that excludes the use of energy-dependent quality control pathways. Our study identified a quality control pathway that lowers the burden of OMPs with a deregulated pore activity in the periplasm by restricting their SecY-mediated translocation.

Bacterial outer membrane porin proteins are beta-barrel-forming proteins that are efficiently translocated through SecY into the periplasm. In addition to their usually 16 beta-strands, porins in *E. coli* contain several short alpha-helical domains, with the first helix being highly conserved (UniProtKB P06996, P02932, P02931). Based on our previous findings that alpha-helical domains increase SecY and Sec61 translocation efficiency of clients dominated by intrinsically disordered domains or beta strands^31,33–35^, we analyzed whether OmpC and OmpF variants lacking helix 1 show a reduced periplasmic localization. Indeed, our analysis revealed a marked reduction of the ∆H1 variants compared to the corresponding wildtype proteins. By performing domain swapping experiments and a mutational analysis we found that the decisive factor for the impaired translocation was not the lack of alpha-helical structure but the loss of a highly conserved motif of negatively charged amino acids. Replacing four aspartates and one glutamate by alanines (OmpC-5A) had the same effect as deleting helix 1. This motif is the central element of the constriction zone or eyelet of the channel^2^. Interaction of this motif with opposing positively charged amino acids in the wall of the beta-barrel is critically dependent to determine pore diameter^48^, ion conductance and gating^6,49–51^, substrate specificity^52,53^, and transport of antibiotics^8,54^. Notably, mutations in this loop do not interfere with the formation of the beta-barrel structure. Indeed, it was shown that these variants can be inserted into artificial membranes in vitro^50^.

Based on these findings it is tempting to speculate that a quality control pathway for variants with mutations in the eyelet should control translocation of such variants into the periplasm in order to prevent insertion of deregulated channels into the OM. Indirect support for this concept was provided by the analysis of an OmpC variant lacking part of the C-terminal domain. OmpC-254X, which consists only of amino acids 1–253 of OmpC, was present in the periplasm, in contrast to the OmpC-254X mutant that in addition has a mutated eyelet motif (OmpC-254X/5A). Thus, it is unlikely that a variant with a mutant eyelet motif (OmpC-5A) is more efficiently subjected to a quality control pathway in the periplasm than a variant lacking the C-terminal third of the protein. Indeed, OmpC-5A was neither secreted via outer membrane vesicles nor stabilized in *E. coli* strains devoid of proteases DegP or FtsH. These results indicated that either targeting of OmpC-5A to SecY was compromised or the subsequent translocation into the periplasm. Our split GFP tagging approach in combination with subcellular fractionation revealed an at least transient interaction of the N-terminal GFP moiety of OmpCΔH1 with the periplasmically localized GFP moiety. We therefore concentrated on a possible role of SecY in controlling periplasmic localization of OMPs with a deregulated channel activity. To this end, we made use of the extensively characterized *prlA4* strain of *E. coli*^25^ that expresses a SecY variant with two amino acid substitutions (F286Y and I408N)^41^. As a consequence of these mutations, the proof-reading capacity of SecY is decreased and translocation of pre-proteins with a defective or missing signal sequence is enabled^42–44^. Mechanistically, the amino acid substitutions in transmembrane segment 7 (F286Y) and 10 (I408N) seem to promote a translocation-competent conformation of the translocon in the absence of a signal peptide^46,47^. Indeed, the eyelet-mutant OmpC variant, which was barely translocated through wildtype SecY, was translocated into the periplasm of the *prlA4* strain with an efficiency similar to that of wildtype OmpC. Although we do not know exactly at which point translocation of mutant OmpC fails, the split GFP protein tagging approach and subcellular fractioning by ultracentrifugation indicated that the translocation processes is halted after signal peptide cleavage of the mutated OmpC variants. This is compatible with the location of the conserved motif 86 amino acids downstream of the signal peptide cleavage site. Testing translocation efficiency of OmpC variants with continuously increased numbers of mutated amino acids revealed a threshold on translocation for variants lacking three or more charged amino acids. Conceptually, the negatively charged amino acids within this motif might provide an additional driving force when the motif is inside the SecY channel, assisting the driving force exerted by SecA. This could be mediated by the proton gradient across the inner membrane or by direct interactions with SecY or translocon-associated factors, like the periplasmic chaperones PpiD or Skp^55,56^. Strikingly, a possible role of a negatively charged motif on nascent chain translocation through SecY has been shown for co-translational translocation^57^. In their study, authors analyzed a pulling force acting on model proteins with negatively charged residues during their passage through the SecY translocon. Their model is based on an electric interaction between negatively charged, grouped residues and the transmembrane potential, enhancing translocation of nascent chains. The conserved motif of negatively charged amino acids we found to be essential for translocation of porins through SecY might therefore enable passing the inner membrane by its overall net charge. Additionally, very recent publications point out a possible role of charged lipids in protein translocation by keeping SecY in an open, translocation competent state^58^. This might be in line with our finding that a *prlA4* strain of *E. coli*, with SecY already locked in an open state, was able to translocate OmpC without the conserved motif of negatively charged amino acids. While the exact mechanism awaits further clarification, our study revealed a novel quality control pathway that protects the integrity and functionality of the OM.

## Experimental procedures

### Constructs/plasmids

Plasmid amplification and maintenance was carried out in *Escherichia coli* TOP10^©^ (ThermoFisher Scientific). All OmpC and OmpF mutants are based on NCBI Gen ID 946716 and 945554, respectively. Constructs include a C-terminal HA-tag, allowing detection by a monoclonal antibody. All constructs used in this study were generated by standard PCR cloning techniques. OmpCΔH1 and OmpFΔH1were created by deleting aa 107–135 and aa 116–144, respectively. OmpCαPrP was created by replacing aa 107–135 of OmpC with aa 200–227 of mouse PRNP (GenBank accession number M18070). Constructs carrying the 1A, 2A, 3A, 4A and 5A mutation were created by inserting point mutations D110A, D120A, D126A, E130A and D134A, respectively. For the fluorescence complementation assays (see below) the signal peptide of DsbA (NCBI Gene ID 948353) was fused to the N-terminus of GFP1-10. The 15 aa GFP11 was inserted into OmpC constructs between aa 21/22 or attached to the C-terminus, respectively.The constructs described above were cloned into pET27b(+) expression vector (Novagen) or pBAD24^59^.

### Antibodies and reagents

The following antibodies were used: anti-HA (mAb, MMS-101R; Covance), anti-MBP-Probe (mAb, sc-32747; SantaCruz), anti-HSP60 (pAb, SC-1052; SantaCruz), IR-Dye conjugated secondary antibody (IR-Dye 800CW Donkey Anti-Mouse/Anti-Goat; Licor). All standard chemicals and reagents were purchased from Sigma-Aldrich if not otherwise noted.

### Translocation studies in *Escherichia coli*

Constructs were cloned into pET27b( +) (Novagen) or pBAD24^59^ and transformed into the *E. coli* expression strains listet (Table 1). Main expression cultures were inoculated from overnight culture to an optical density of OD_600 nm_ = 0.05 and grown in LB substituted with 50 µg/mL kanamycin (pET27b(+)) or 100 µg/mL ampicillin (pBAD24) at 30 °C, 120 rpm. Protein expression was induced at OD_600 nm_ = 0.5 for 2 h by addition of 1 mM IPTG or 0.05% l-Arabinose. The isolation of the periplasmic fraction is based on a method described previously^35^. In brief, 10 ml of main culture were harvested by centrifugation at 4000*g* for 1 min at 4 °C. The cell pellet was carefully resuspended in TSE Periplasmic Extraction buffer (200 mM Tris–HCl, pH 8.0; 500 mM sucrose, 1 mM EDTA, cOmplete^®^ Mini EDTA-free Protease Inhibitor Cocktail (Roche)) using a wire loop. Respective volume of TSE for resuspension was calculated according to OD_600 nm_ of the main culture at the time of harvesting (OD_600 nm_ = 1.0 ≈ 1.000 µl TSE buffer). After 30 min incubation on ice, cells were centrifuged for 30 min at 21,000*g* and 4 °C. Supernatant containing the periplasmic fraction was isolated for Western blotting. The cell pellet was resuspended in TE-buffer (same volume as TSE buffer, 10 mM Tris–HCl, 1 mM EDTA, pH 8, cOmplete^®^ Mini EDTA-free Protease Inhibitor Cocktail (Roche)), and cells were opened by sonification and again centrifuged for 30 min at 21,000*g* and 4 °C to obtain soluble cytoplasmic fraction for Western blotting.

**Table 1:** *Escherichia coli* strains used for expression/translocation studies.

Strain	Genotype	References
BL21 (DE3)	F–, *ompT gal dcm lon hsd*S_B_(*r*_B_^-^ *m*_B_^-^) λ(DE3 [*lacI lacUV5-T7p07 ind1 sam7 nin5*]) [*malB*^+^]_K-12_(λ^S^)	^62^
BW25133 ΔDegP	F-, *Δ(araD-araB)567*, *ΔdegP775::kan*, *ΔlacZ4787*(::rrnB-3), *λ*^*−*^, *rph-1*, *Δ(rhaD-rhaB)568*, *hsdR514*	^63^
Δ*ftsH* (AR3291)	F- IN(*rrnD-rrnE*)1, Δ*ftsH3*::*kann, sfhC21, zad220*::Tn*10*	^64^
MC4100	F^−^, [*araD139*]_B/r_, Δ(*argF-lac*)169, *λ*^−^, *e14*-, *flhD5301*, Δ(*fruK-yeiR*)725 (*fruA25*), *relA1*, *rpsL150*(*strR*), *rbsR22*, Δ(*fimB-fimE*)632(::IS*1*), *deoC1*	^65^
MC4100 *prlA4*	*secY(prlA4)*	^25^

### Western blotting

For Western blot analysis, lysates were boiled in Laemmli sample buffer with ß-mercaptoethanol (4% (v/v)). Following SDS-PAGE proteins were transferred to nitrocellulose by electroblotting (Mini Trans-Blot^®^ Cell, BioRad). Membranes were blocked by incubation in TBS-T (TBS with 0.1% (w/v) Tween 20) containing 5% skimmed milk for 1 h at room temperature and incubated with primary antibody in TBS-T + 5% skimmed milk overnight at 4 °C. After washing with TBS-T blots were incubated with respective secondary antibody (IRDye-Infrared Technology Licor) in TBS-T for 1 h at room temperature. Protein signals were visualized using ODYSSEY^®^ 9120 Scanner and Image Studio Light software (Licor).

### Pulse chase experiments

*Escherichia coli* BL21 DE3 cells producing OmpC or the OmpC-5A variant from the pET27b plasmid were grown in M63 minimal medium containing 18 amino acids (except methionine and cysteine) until OD_600_ = 0.5–0.6. As a control BL21 DE3 cells without plasmid were used. 1 mM IPTG was added for *T7* RNA polymerase induction for 15–60 min at 37 °C. Subsequently, 50 µg/µl of rifampicin (final concentration) was added and cells were incubated for 10 min at 37 °C. The optical density of the culture was measured and 10^8^ cells were transferred to 2 ml reaction tubes and the volume was adjusted to 2 ml with M63 medium. Then 2 µl of ^35^S –Met/Cys labeling mix (Perkin Elmer) were added to each sample. After 5 min of incubation, 100 µl of sample were taken from each reaction and directly precipitated with 5% trichloroacetic acid (TCA). Non-radioactive Met/Cys mix (1 µg/ml final concentration) was then added to start the chase. 100 µl samples were taken after several time points and directly precipitated with 5% TCA. Sample were centrifuged at 13,500 rpm for 15 min at 4 °C, the pellet was dissolved in SDS loading dye and denatured at 56 °C for 10 min. Samples were separated by a 15% SDS-PAGE gel and radioactivity was quantified by phosphorimaging using a Typhoon Phosphorimager (GE Healthcare). Analyses were performed by using the *ImageJ* software and by setting the total activity present in signal sequence containing band and the processed band to 100%. Percent processing was then calculated. Quantification reflects at least two biological replicates with two technical replicates each. The error bars represent the standard error of the mean (SEM), which were calculated by the *MS Excel* software.

### Isolation of outer membrane vesicles

For isolation of *Escherichia coli* outer membrane vesicles 10 ml culture medium was collected and separated from cells by centrifugation (1 min, 4 °C, 5000*g*) and subsequent filtering through a 0.4 µm sterile filter. Flow through was centrifuged for 4 h at 100,000*g*, the resulting pellet resuspended in 100 µl TE buffer and analyzed via SDS-PAGE and subsequent Western Blotting.

### Membrane fractionation of *E. coli*

Fractionation of *E. coli* inner membrane is based on sucrose gradient ultracentrifugation as described before^60,61^. In brief, *E. coli* cells from mid-exponential phase were harvested, lysed by French pressing and centrifuged at 30,000*g*. The supernatant was further centrifuged at 150,000*g* for 2 h and the pellet was resuspended in 50 mM triethanolamine acetate, pH 7, 5, 250 mM sucrose, 1 mM DTT (INV buffer) and loaded on a three-step sucrose gradient, containing 0.77 M sucrose, 1.44 M sucrose and 2.02 M sucrose, each prepared in INV buffer. After centrifugation at 82,000*g* in a swing-out rotor (Sorvall AH629) for 16 h, the inner membrane fractions were removed from the gradient, diluted in INV buffer, pelleted by centrifugation (150,000*g*, 2 h) and resuspended in INV buffer.

### Fluorescence complementation assays

Complementing constructs were cloned into pET27b(+) (Novagen) and pBAD24^59^ and transformed into the *E. coli* BL21 (DE3). Main expression cultures were inoculated from overnight cultures to an optical density of OD_600 nm_ = 0.05 and grown in LB substituted with 50 µg/mL kanamycin and 100 µg/mL ampicillin at 30 °C, 120 rpm. Protein expression of GFP1-10 was induced at OD_600 nm_ = 0.4 for 2 h by addition of 0,05% l-Arabinose, followed by addition of 1 mM IPTG for expression of OmpC constructs for 1 h. Cells were harvested by centrifugation (1 min at 5000*g*), washed with PBS and fixated with 10% paraformaldehyde for 10 min on ice. After two times washing with PBS cells were spread on a glass cover slip and analyzed via super-resolution structured illumination microscopy (SR-SIM) using a Zeiss ELYRA PS.1 and ZEN Black 2.3 software.

### Quantification and statistical analysis

Data represent the mean plus/minus SEM from n = 5 biological replicates. All statistical analyses were performed by using GraphPad Prism (version 5; San Diego, CA, USA). To analyze the distribution of datasets (parametric/non-parametric), Kolmogorov–Smirnov test were performed. For the comparison of more than two parametric datasets, one-way ANOVA was used. To correct for α-error inflation resulting from multiple comparisons, ANOVA Dunnett’s post hoc multiple comparison test was performed.

## Supplementary information

Supplementary Information.
