# Supplementary material for: SecY-mediated quality control prevents the translocation of non-gated porins
Source: Sci Rep. 2020 Oct 1;10:16347. doi: 10.1038/s41598-020-73185-y (PMC7530735; doi:10.1038/s41598-020-73185-y)

SecY-mediated quality control prevents the translocation of non-gated porins

Sebastian Jung[1], Verian Bader[1*], Ana Natriashvili[2*], Hans-Georg Koch[2], Konstanze F. Winklhofer[3] and Jörg Tatzelt[1#]

[1]Department Biochemistry of Neurodegenerative Diseases, Institute of Biochemistry and Pathobiochemistry, Ruhr University Bochum, Germany; [2]Institute of Biochemistry and Molecular Biology, ZBMZ, Faculty of Medicine, Albert-Ludwigs-University Freiburg, Germany; [3]Department Molecular Cell Biology, Institute of Biochemistry and Pathobiochemistry, Ruhr University Bochum, Germany

Supplementary Figure 1

Figure 1B, OmpC

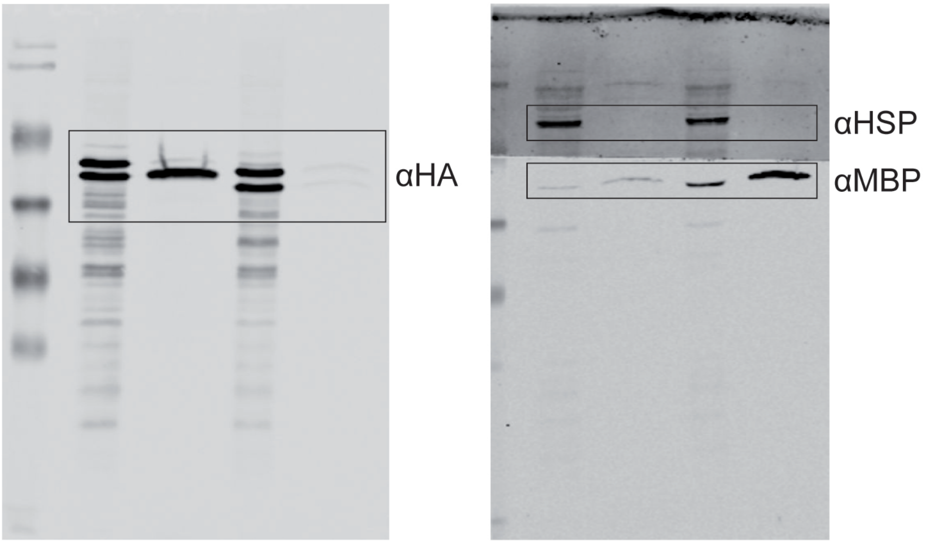

Figure 1B, OmpF

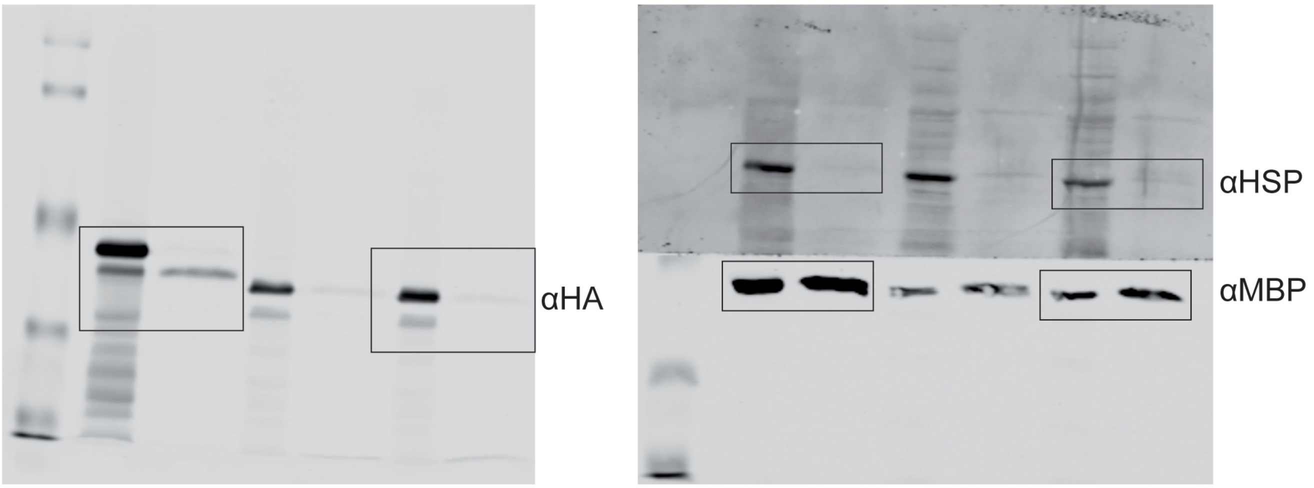

Figure 1B, OmpC Cytosol

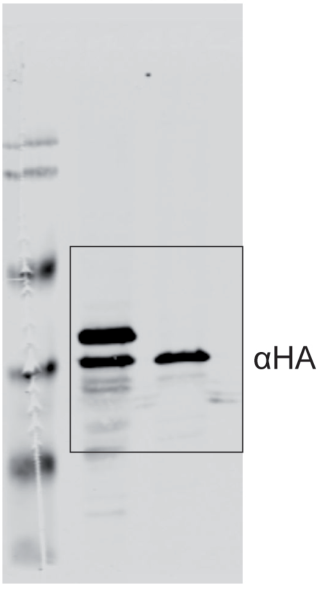

Figure 1D, OmpC-254X

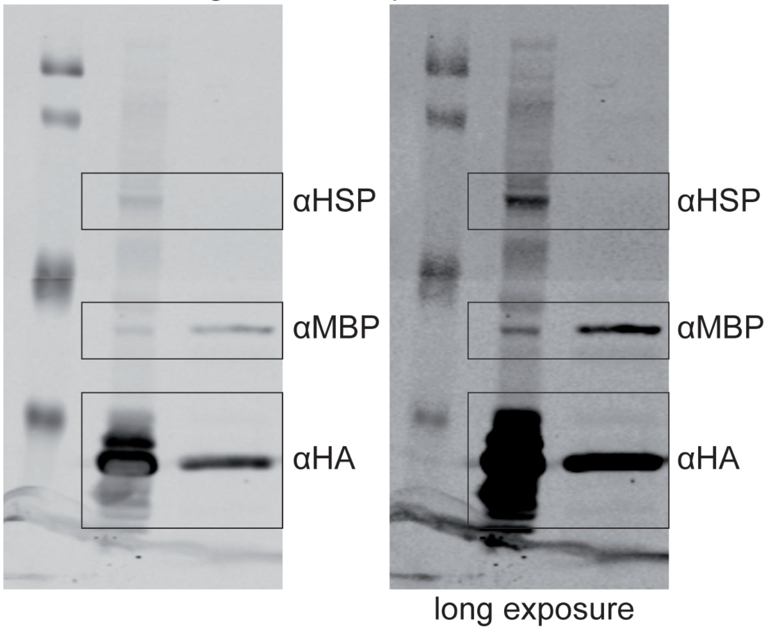

Supplementary Figure 2

Figure 1F

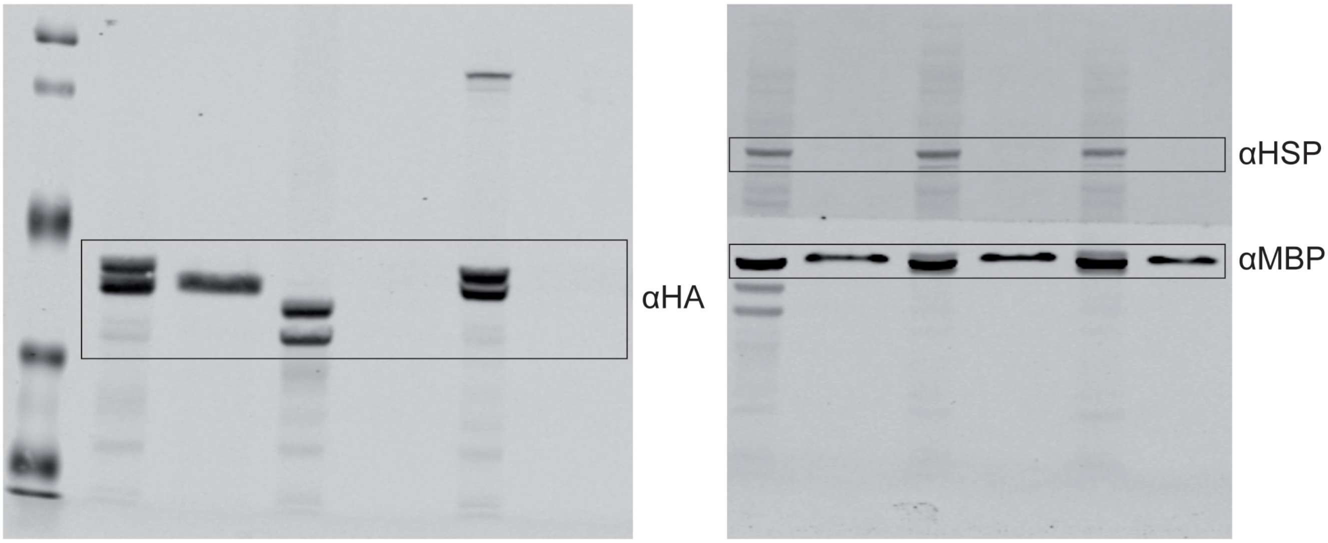

Figure 2C

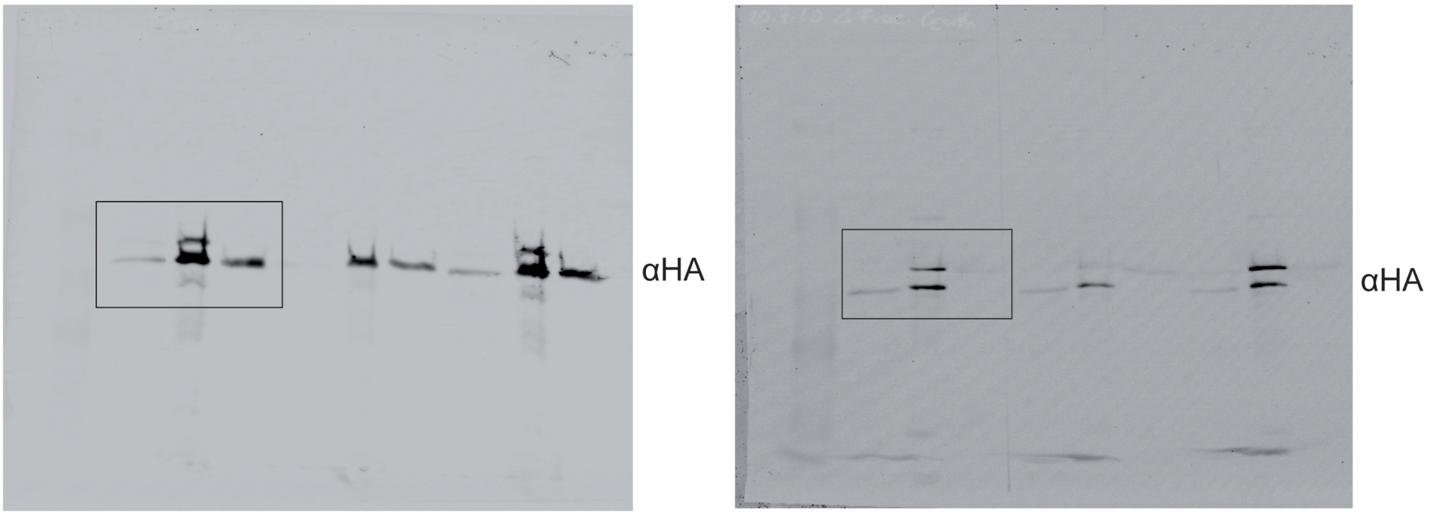

Supplementary Figure 3

Figure 3B, OmpC WT, 5A

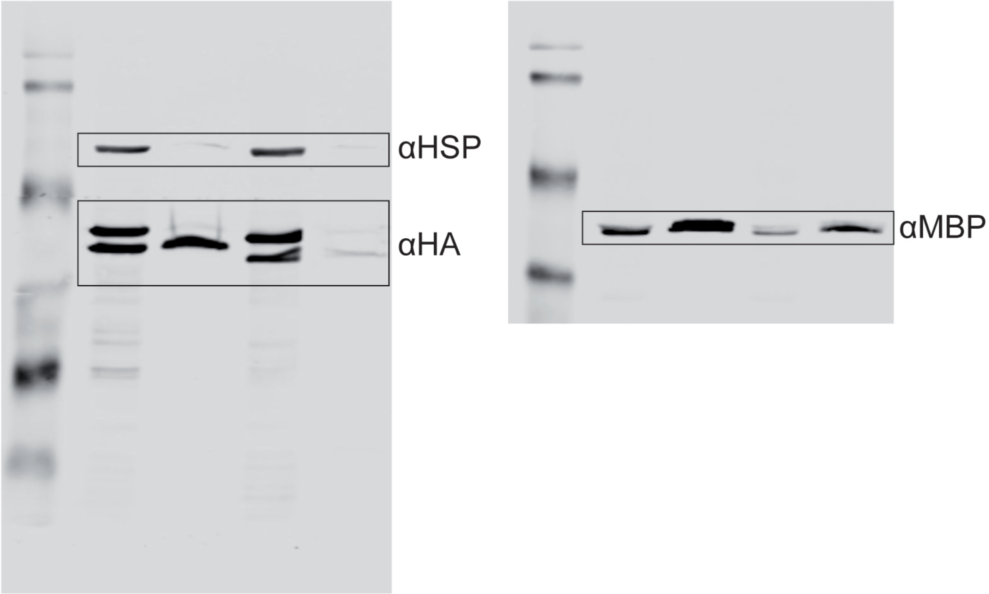

Figure 3B, OmpC 254X, 254X/5A

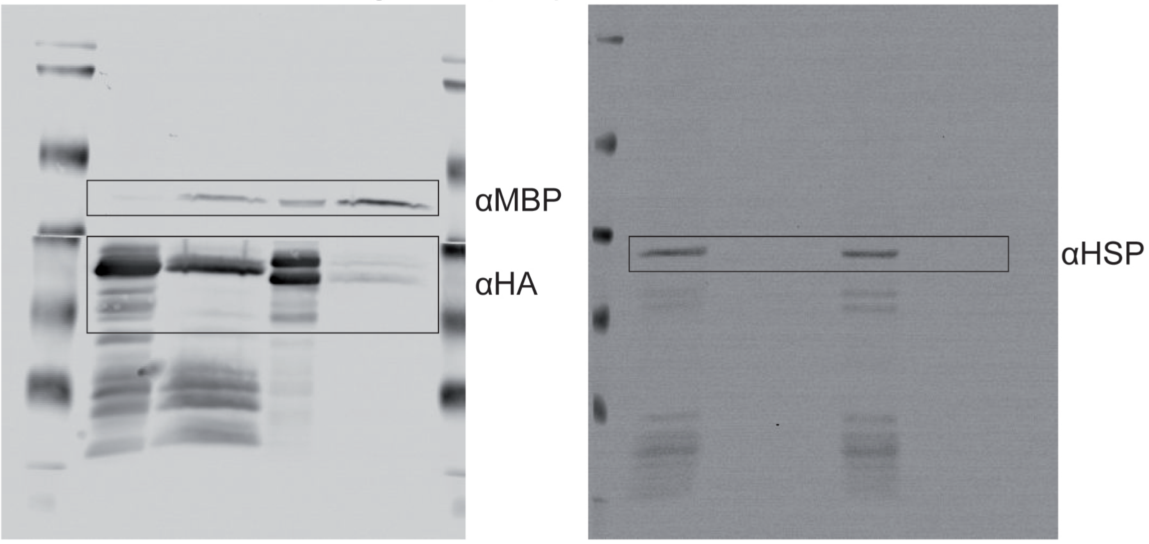

Figure 3C Pulse Chase

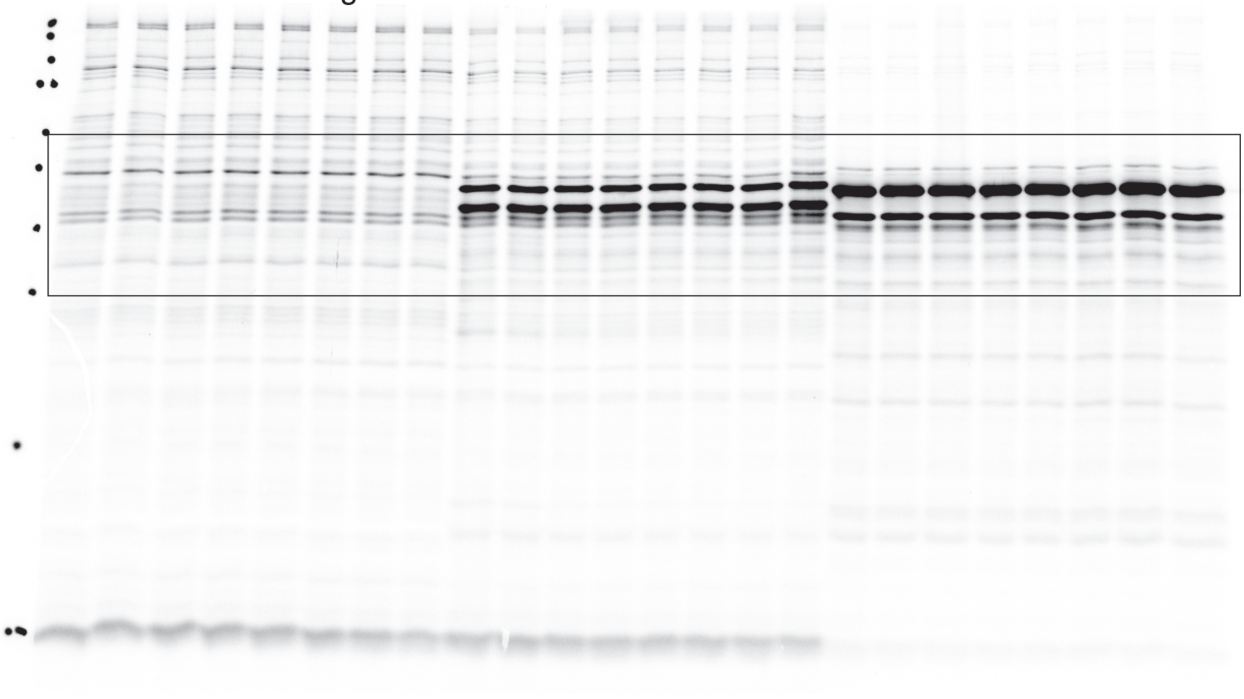

Supplementary Figure 4

Figure 4

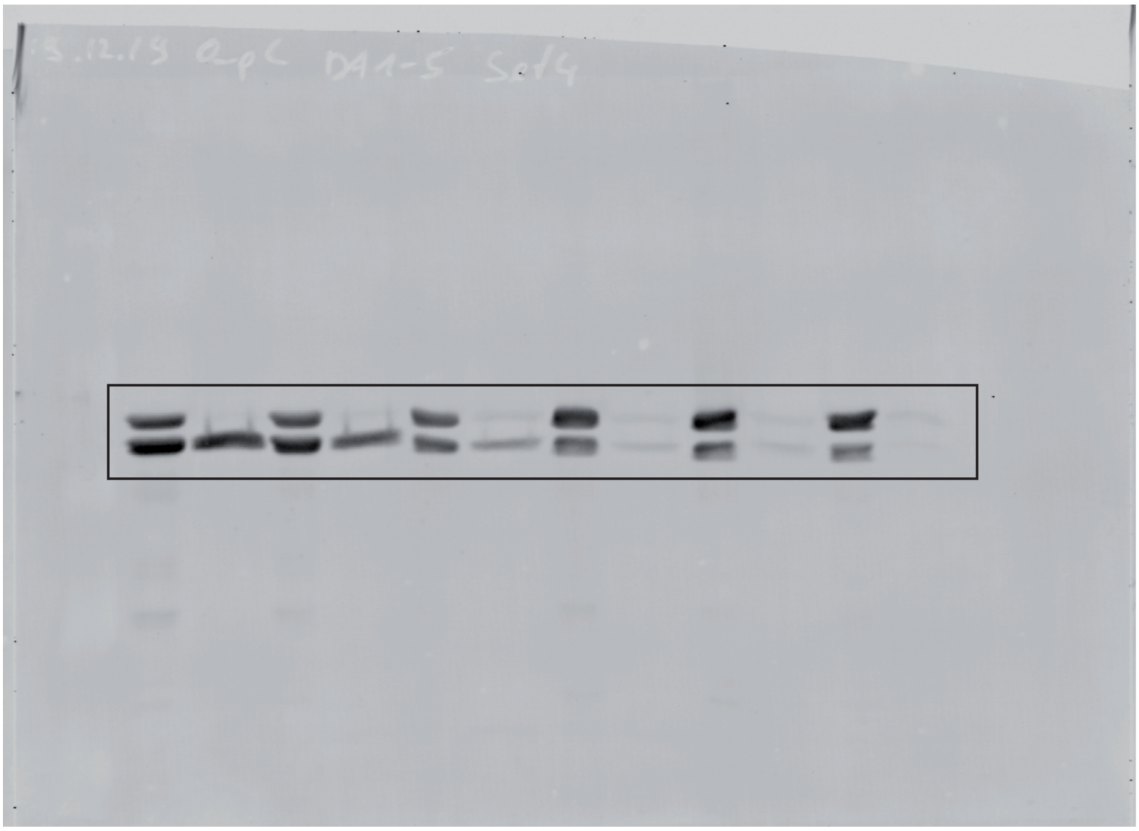

Supplementary Figure 5

Figure 5A, BW25133 ΔDegP

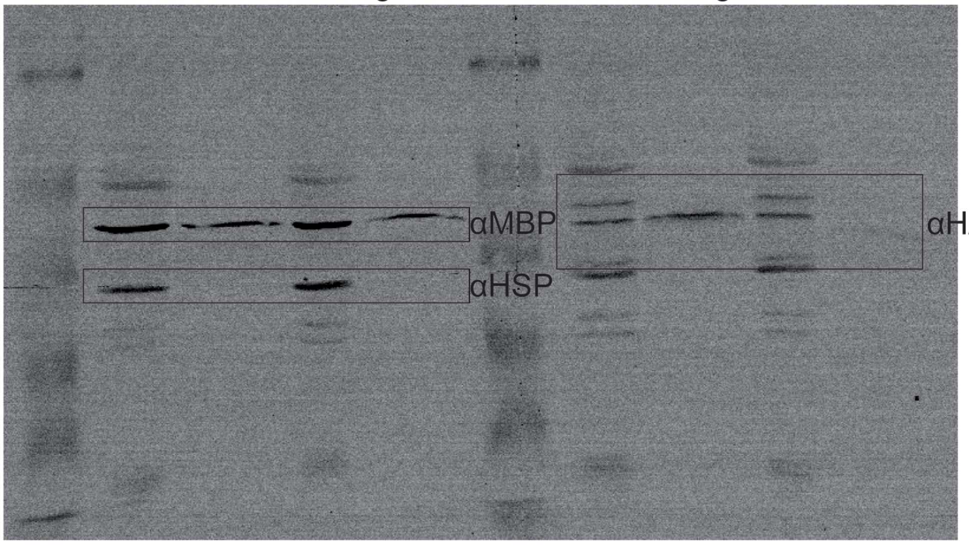

Figure 5A, W3110 ΔFtsH

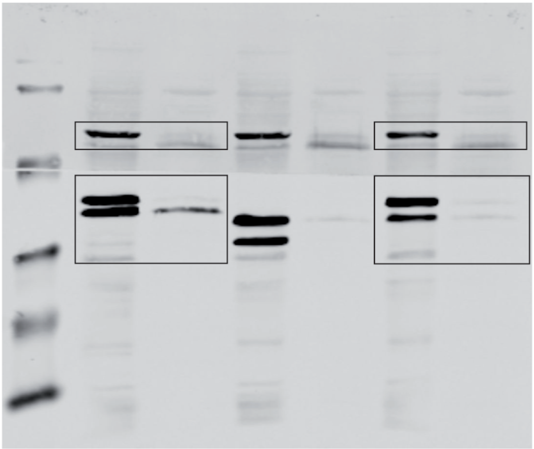

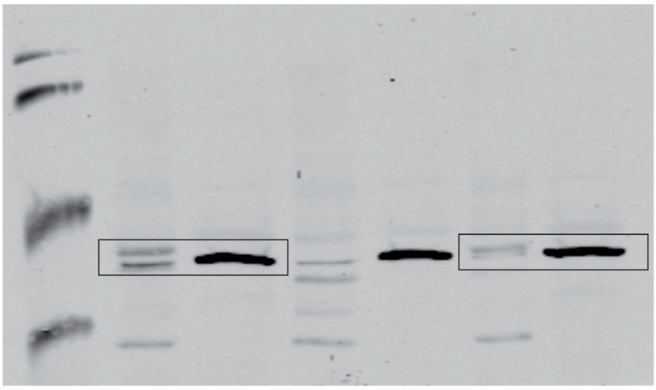

Figure 5b, OmpC OMV

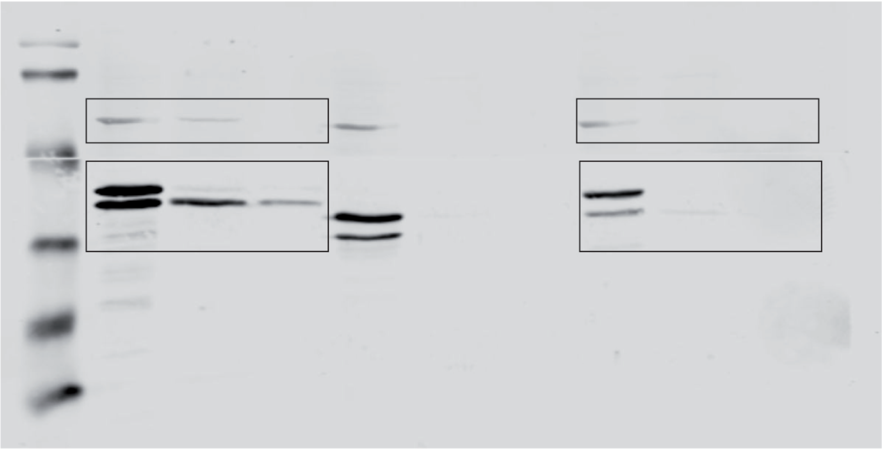

Supplementary Figure 6

Figure 5C, MC4100

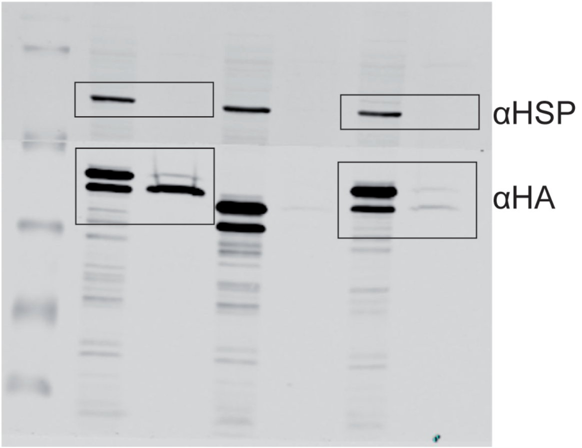

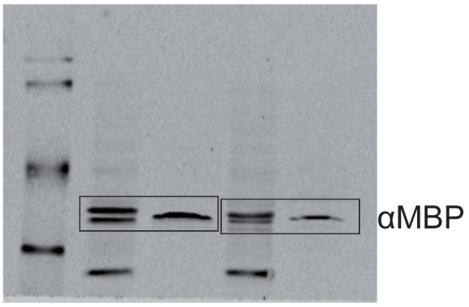

Figure 5C, MC4100 *prlA4*

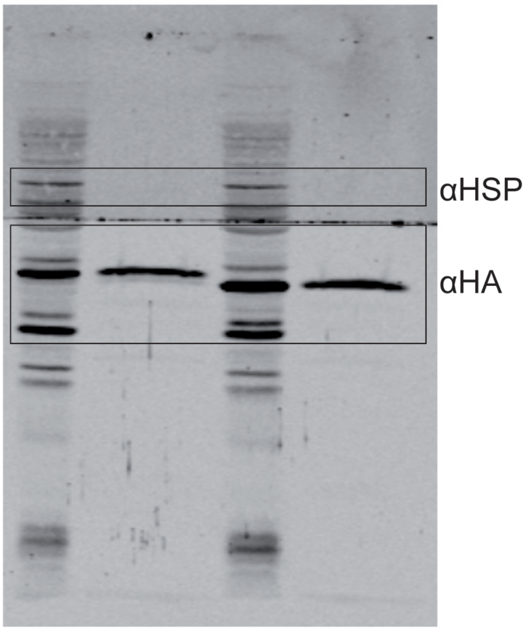

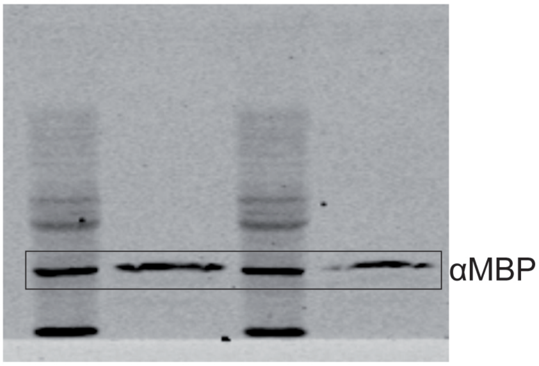

Supplement: Supplementary file 1 — Supplementary Information. [file 41598_2020_73185_MOESM1_ESM.pdf]
